# Supplementary material for: IL-17-producing γδ T cells switch migratory patterns between resting and activated states
Source: Nat Commun. 2017 Jun 5;8:15632. doi: 10.1038/ncomms15632 (PMC5465362; doi:10.1038/ncomms15632)
Supplement: Supplementary Information — Supplementary Figures and Supplementary Tables [file ncomms15632-s1.pdf]

SUPPLEMENTARY INFORMATION

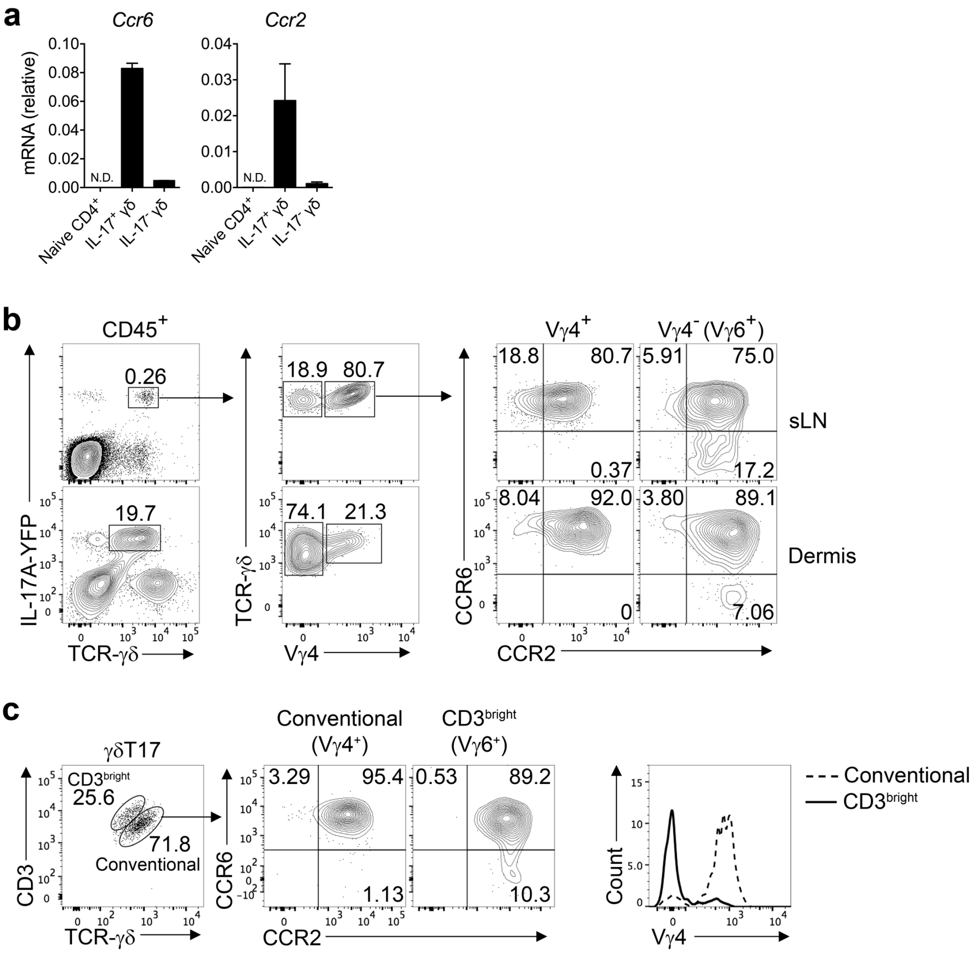

Supplementary Figure 1: CCR6 and CCR2 expression by resting γδT17 cells. a) *Ccr6* and *Ccr2* mRNA in sorted $CD3^{+}CD4^{+}CD44^{lo}CD25^{-}$ naïve CD4 T cells and $CD90^{+}CD3^{+}$TCR-$\gamma\delta^{+}$IL-17A-YFP^{+} γδT17 and YFP^{-} IL-17^{-} γδ T cells from skin-draining lymph nodes (sLNs) and spleen of naïve $Il17a^{Cre}$×$Rosa26^{eYFP}$ mice (pooled from n=5). N.D.: not detected. **b)** Representative flow cytometry of CCR6/CCR2 expression by $V\gamma4^{+}$ and $V\gamma4^{-}$ ($V\gamma6^{+}$) γδT17 cells from sLNs and dermis of naïve $Il17a^{Cre}$×$Rosa26^{eYFP}$ mice (n=3). **c)** Representative flow cytometry of CCR6/CCR2 and Vγ4 expression by conventional ($V\gamma4^{+}$) and $CD3^{bright}$ ($V\gamma6^{+}$) γδT17 cells from sLNs of naïve $Il17a^{Cre}$×$Rosa26^{eYFP}$ mice (n=3). Mean±SD. **b-c)** Representative of two experiments.

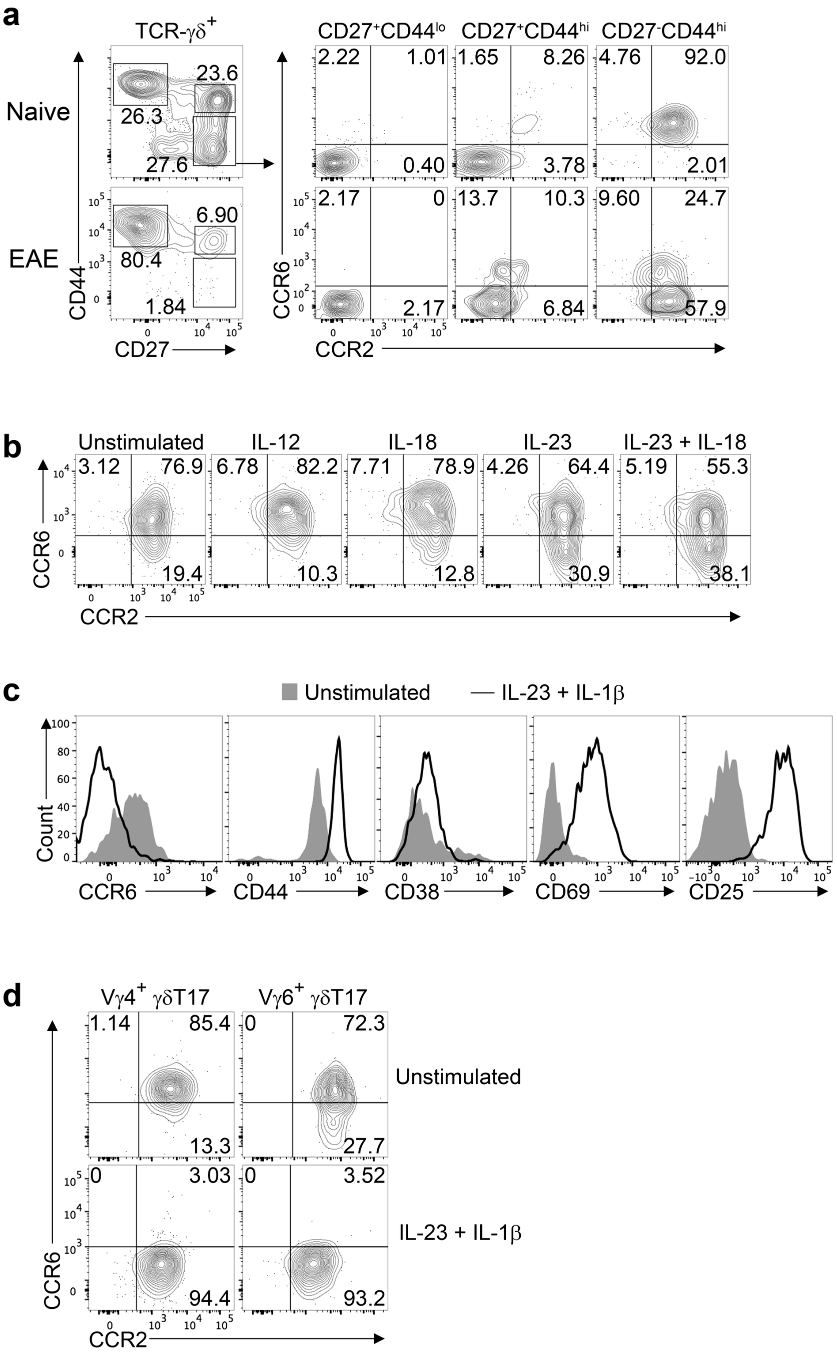

Supplementary Figure 2: Downregulation of CCR6 in activated γδT17 cells. a) Representative flow cytometry of CCR6/CCR2 expression by subsets of γδ T cells distinguished by CD44 and CD27 expression in skin-draining lymph nodes of wildtype (WT) mice either naïve or at experimental autoimmune encephalomyelitis (EAE) onset (n=3/group). **b)** Representative flow cytometry of CCR6/CCR2 expression by γδT17 cells from *Il17a*^{Cre}x*Rosa26*^{eYFP} lymphocytes cultured with indicated stimuli for 72 hr (n=3). **c)** Representative flow cytometry of activation marker expression by γδT17 cells from *Il17a*^{Cre}x*Rosa26*^{eYFP} lymphocytes either unstimulated or IL-23/IL-1β stimulated for 72 hr (n=3). **d)** Representative flow cytometry of CCR6/CCR2 expression by splenic conventional (Vγ4^{+}) and CD3^{bright} (Vγ6^{+}) γδT17 cells (gated as in Supplementary Figure 1c) from *Il17a*^{Cre}×*Rosa26*^{eYFP} mice either unstimulated or IL-23/IL-1β stimulated for 72 hr (n=3). **a, d)** Representative of two experiments.

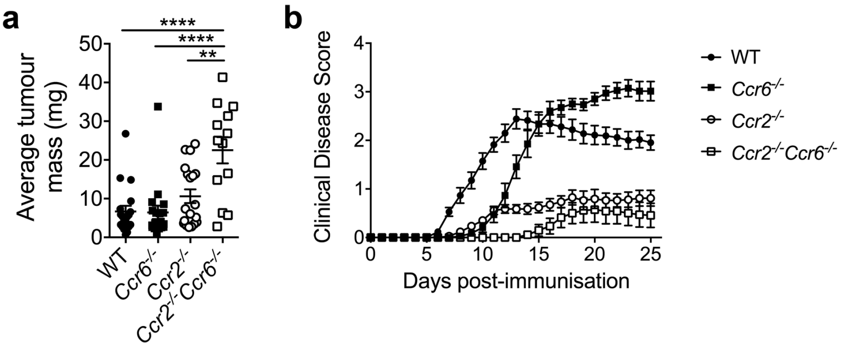

Supplementary Figure 3: B16 melanoma and EAE progression in chemokine receptor-deficient mice. a) Average mass (per mouse) of B16 melanomas 7d post-challenge in wildtype (WT) (n=19), *Ccr6*$^{-/-}$ (n=18), *Ccr2*$^{-/-}$(n=19) and *Ccr2*$^{-/-}$*Ccr6*$^{-/-}$ mice (n=13). b) Clinical disease scores of WT, *Ccr6*$^{-/-}$, *Ccr2*$^{-/-}$ and *Ccr2*$^{-/-}$*Ccr6*$^{-/-}$ mice given experimental autoimmune encephalomyelitis (EAE) (n=17/group). Mean±SEM. **a-b)** Pooled from two experiments. **a)** One-way ANOVA with Bonferroni's multiple comparisons test. ** $p < 0.01$, **** $p < 0.0001$.

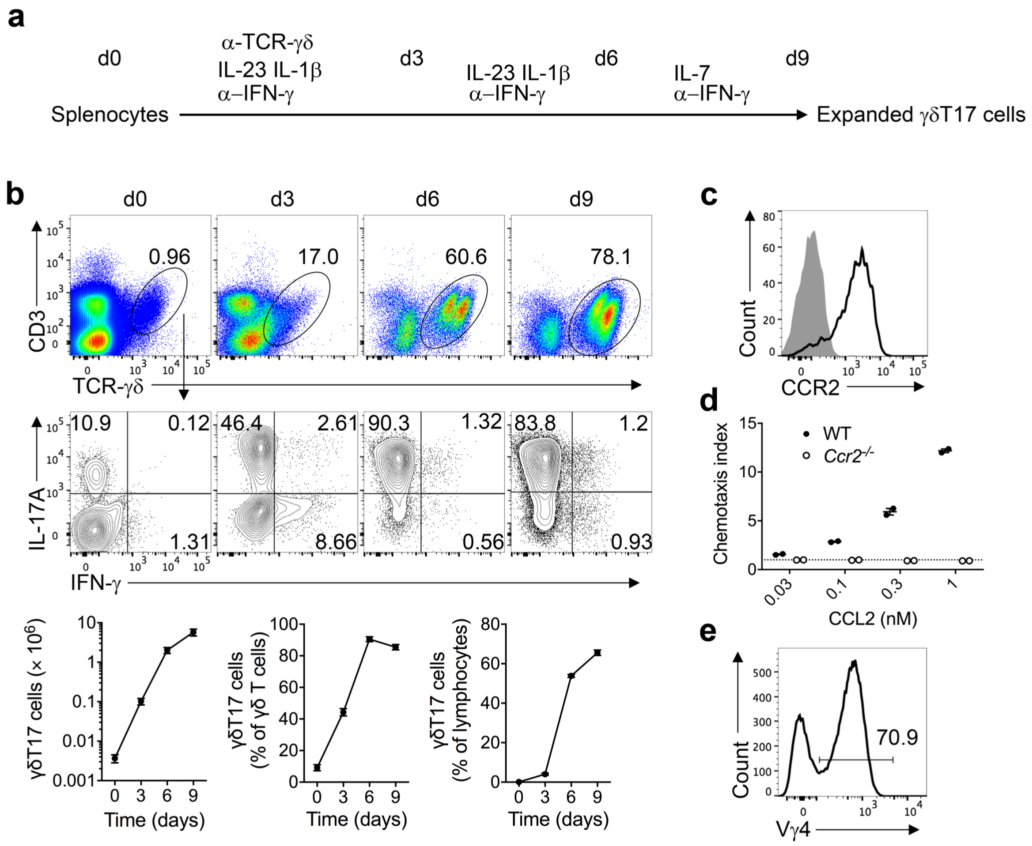

Supplementary Figure 4: *In vitro* expansion of γδT17 cells. a) Schematic of culture protocol. **b)** Representative flow cytometry and number and frequency of *in vitro*-expanded γδT17 cells at different stages of culture (n=3). **c)** Flow cytometry of CCR2 expression by *in vitro*-expanded γδT17 cells relative to isotype (grey). **d)** Transwell chemotaxis of *in vitro*-expanded γδT17 cells from wildtype (WT) and *Ccr2*$^{-/-}$ mice to CCL2. **e)** Flow cytometry of Vγ4 expression by *in vitro*-expanded γδT17 cells. **b)** Mean±SEM, **d)** Mean±SD. **a-e)** Representative of two experiments.

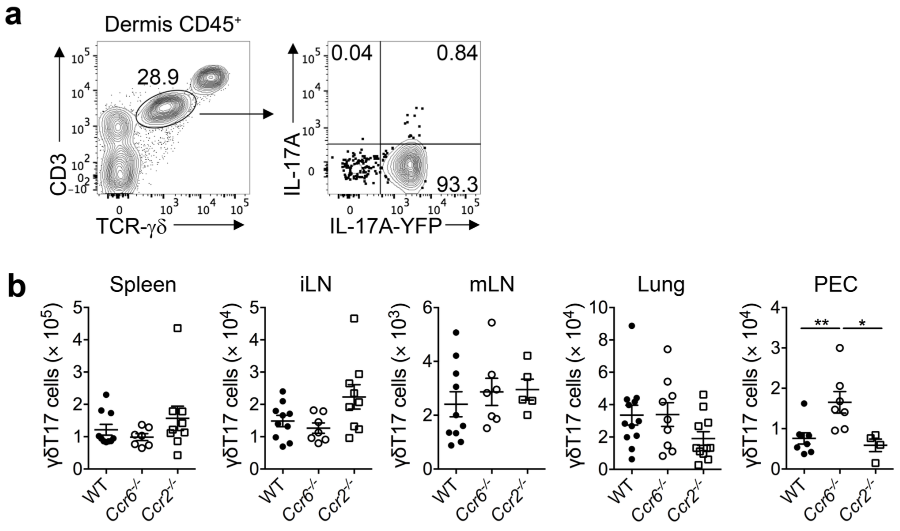

Supplementary Figure 5: CCR6 regulates homeostatic γδT17 cell positioning in the dermis. a) Representative flow cytometry of IL-17A-YFP and IL-17A expression by dermal γδT^{lo} cells from ears of $\textit{Il17a}^{Cre}$x$\textit{Rosa26}^{eYFP}$ mice (n=3). **b)** Number of γδT17 cells in organs of unimmunized wildtype (WT), $\textit{Ccr6}^{-/-}$ and $\textit{Ccr2}^{-/-}$ mice (n=4-12/group). iLN: inguinal lymph node; mLN: mesenteric lymph node; PEC: peritoneal exudate cells. Mean±SEM. **a)** Representative of two experiments, **b)** pooled from two experiments. **b)** One-way ANOVA with Bonferroni's multiple comparisons test. * $p < 0.05$, ** $p < 0.01$.

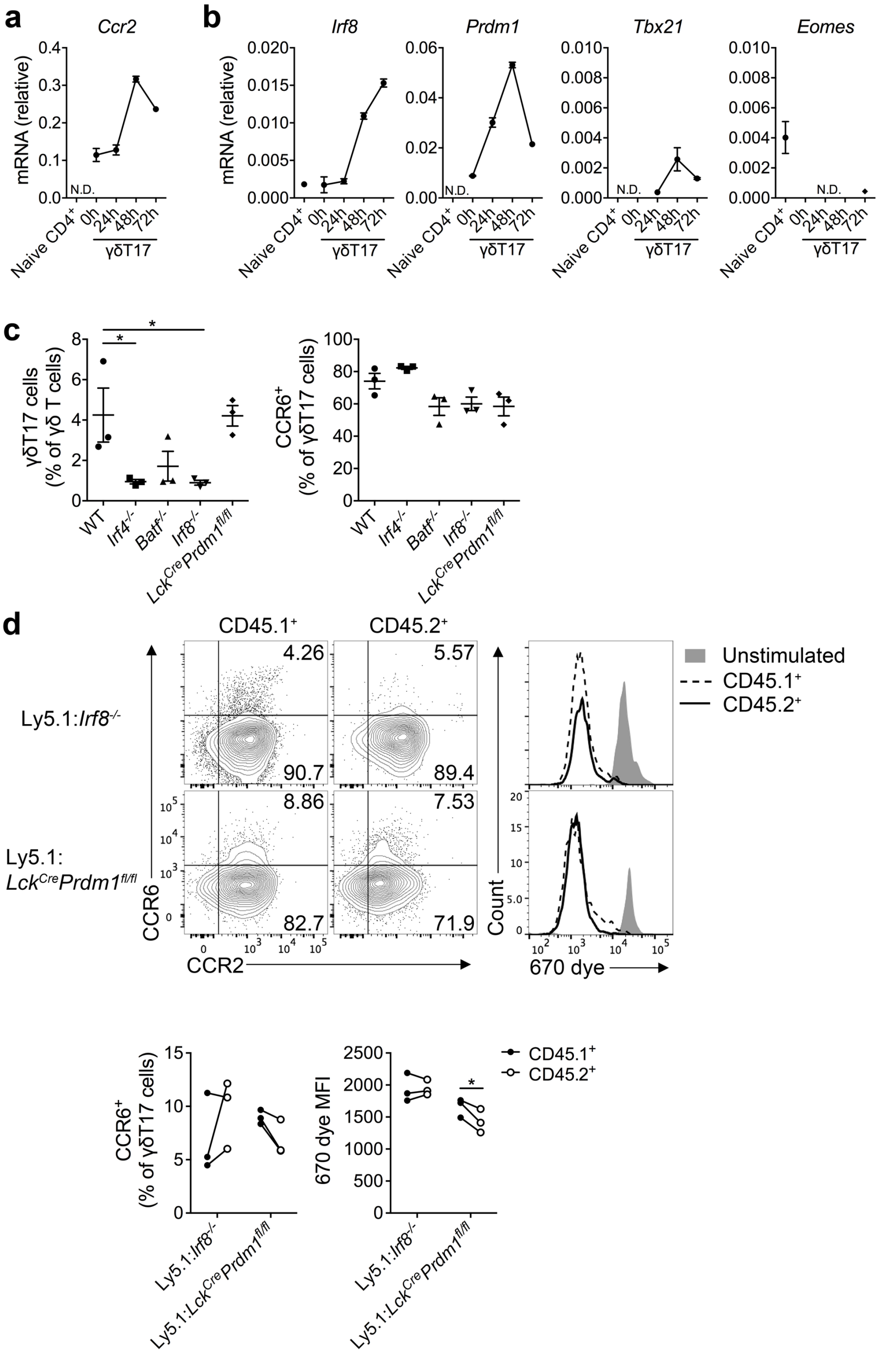

Supplementary Figure 6: IRF8 and Blimp1 do not regulate CCR6 expression in γδT17 cells. a) *Ccr2* and **b)** transcription factor mRNA in sorted γδT17 cells from $Il17a^{Cre}$x$Rosa26^{eYFP}$ lymphocytes fresh *ex vivo* or cultured with IL-23/IL-1β for indicated times (pooled from 5-7 mice). N.D.: not detected. **c)** Frequency of total and $CCR6^{+}$ γδT17 cells in spleens of wildtype (WT) and transcription factor-deficient mice (n=3/group). **d)** Splenocytes from Ly5.1 and $Irf8^{-/-}$ or $Lck^{Cre}Prdm1^{fl/fl}$ mice were 670 dye-labelled, mixed 50:50 and stimulated with IL-23/IL-1β for 72 hr. Representative flow cytometry and quantitation of CCR6 expression and proliferation in $CD45.1^{+}$ or $CD45.2^{+}$ γδT17 cells (n=3/group). **a-b)** Mean±SD, **c)** Mean±SEM. **a-b, d)** Representative of two similar experiments. **c)** One-way ANOVA with Dunnett's multiple comparisons test relative to WT control, **d)** paired two-tailed Student's *t*-test. * $p < 0.05$.

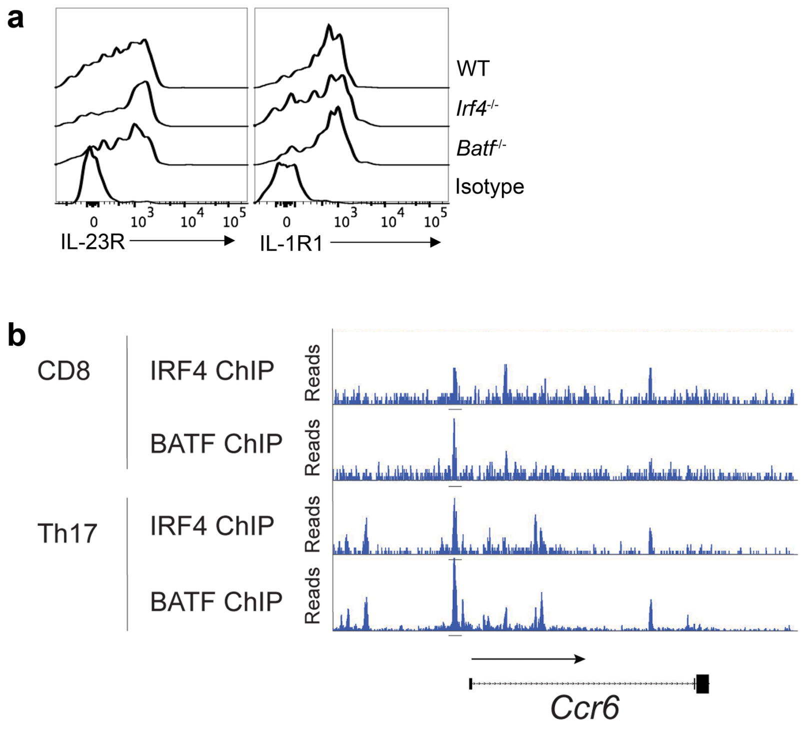

Supplementary Figure 7: IRF4 and BATF promote CCR6 downregulation in γδT17 cells. a) Representative flow cytometry of IL-23R and IL-1R1 expression by splenic CD44^{hi}CD27^{-} γδ T cells (gated as in Supplementary Figure 2a) from wildtype (WT), *Irf4*$^{-/-}$ and *Batf*$^{-/-}$ mice (n=3/group). **b)** IRF4 and BATF ChIP-Seq data at the *Ccr6* locus from CD8^{+} T cells and T helper 17 (Th17) cells from published datasets. Line indicates binding site consistently detected for both transcription factors in both cell types. Datasets are from Kurachi *et al.* 2014 (BATF CD8^{+} T cells), Man *et al.* 2013 (IRF4 CD8^{+} T cells) and Ciofani *et al.* 2012 (IRF4/BATF Th17 cells) as referenced in results section.

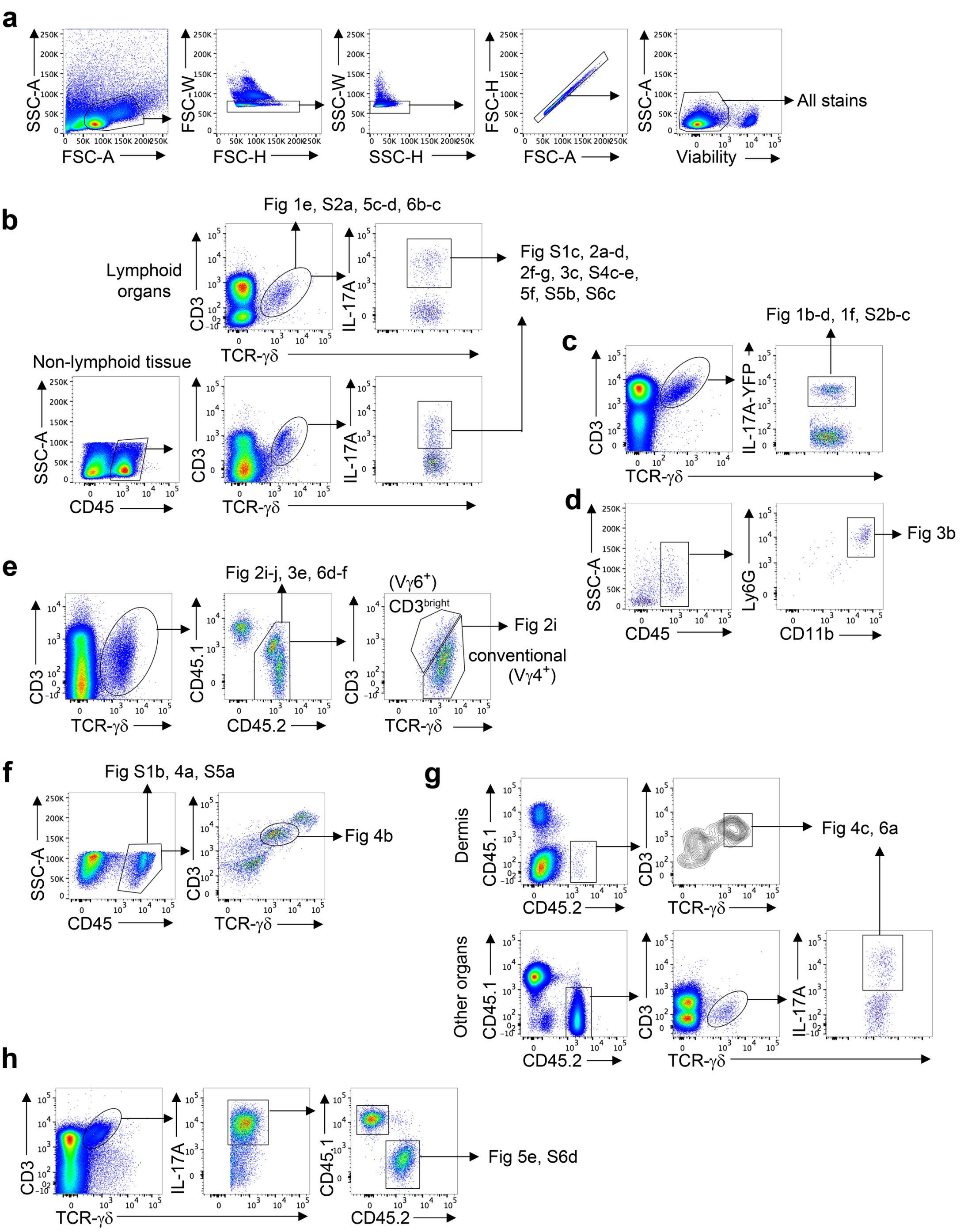

Supplementary Figure 8: Flow cytometry gating strategies. Each panel denotes pre-gates to flow cytometry plots and/or data points in indicated figures. **a)** Lymphocyte gating, single cell discrimination and viability gating serves as pre-gate for all flow cytometry data and sorting strategies. **b)** Gating of γδT17 cells by IL-17A protein expression. **c)** Gating of γδT17 cells in $\mathit{Il17a}^{Cre}\times \mathit{Rosa26}^{eYFP}$ mice. **d)** Gating of neutrophils from nasal wash. **e)** Gating of donor $CD45.1^{+/-}CD45.2^{+}$ *in vitro*-expanded γδT17 cells in co-transfer trafficking experiments, including Vγ4/Vγ6 distinction by $CD3^{bright}$ staining. **f)** Gating of dermal $γδT^{lo}$ cells. **g)** Gating of donor $CD45.2^{+}$ $γδT^{lo}$/γδT17 cells in skin-homing transfer experiments. **h)** Gating of $CD45.1^{+}$ and $CD45.2^{+}$ γδT17 cells in co-culture experiments.

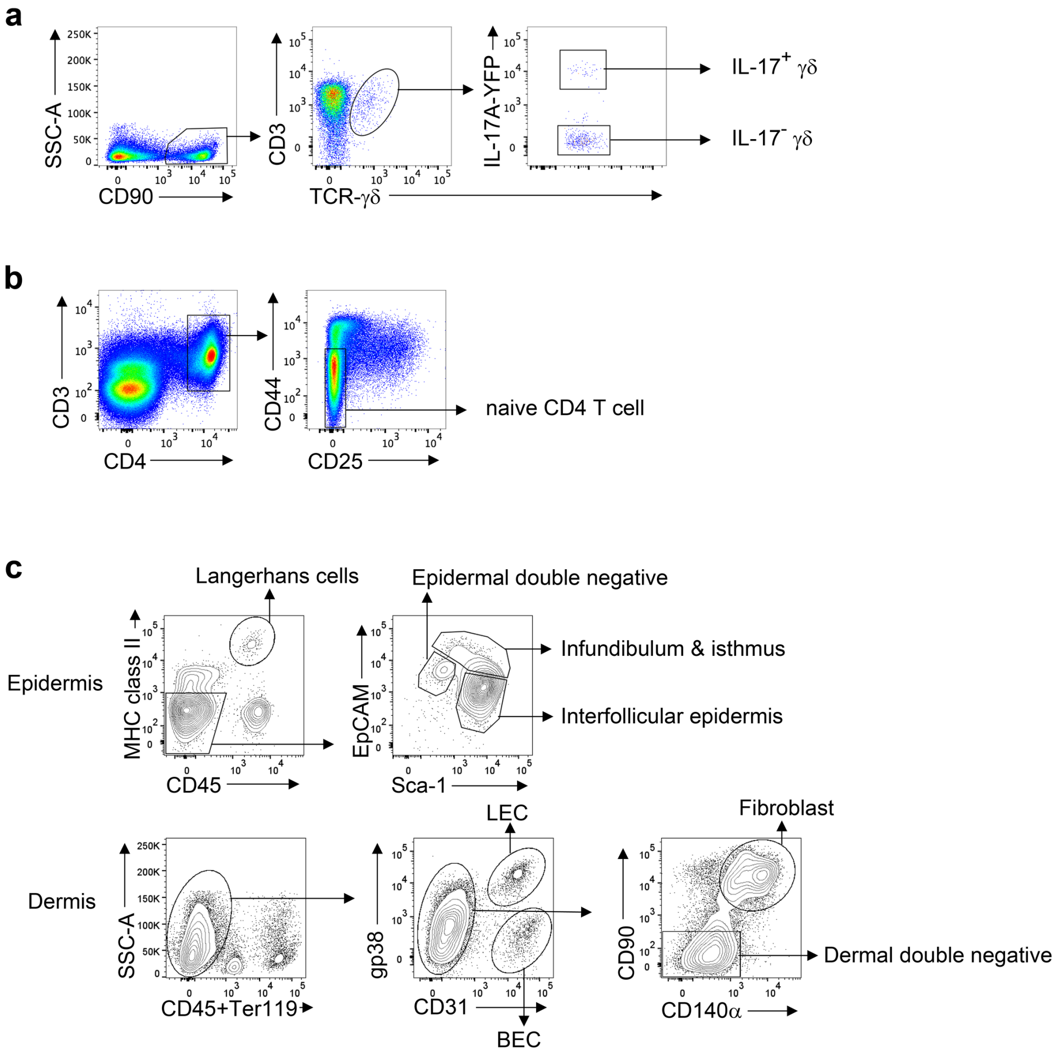

Supplementary Figure 9: Flow sorting strategies. a) Sorting of γδT17 cells and IL-17^{-} γδ T cells from pooled splenocytes and lymph node cells from *Il17a*^{Cre}×*Rosa26*^{eYFP} mice. **b)** Sorting of naïve CD4^{+} T cells from wildtype (WT) splenocytes. **c)** Sorting of indicated epidermal keratinocyte and dermal stromal populations from skin of WT mice. All strategies first gated on live single cells as in Supplementary Figure 8a.

Supplementary Table 1: Monoclonal antibodies and related reagents

**Primary antibodies for flow cytometry, ELISA and cell culture**				
Antigen	Conjugate	Clone	Company	Final concentration
BrdU	FITC	B44	BD	15µL/well
CCL2	Biotin (ELISA)<br>Purified (ELISA)	Polyclonal	R&D<br>R&D	200ng/ml<br>200ng/ml
CCR2	Purified	MC21	In house	5.6µg/ml
CCR6	Purified<br>PE	MAB590<br>140706	R&D<br>R&D	8.3µg/ml<br>7µL/well
CD3ϵ	Biotin<br>BV510<br>FITC<br>PECy7	17A2<br>145-2C11	eBioscience<br>BD<br>BD<br>eBioscience	2µg/ml<br>0.833µg/ml<br>2µg/ml<br>2µg/ml
CD4	Alexa 647	RM4-5	BD	0.67µg/ml
CD11b	PECy7	M1/70	BD	0.67µg/ml
CD25	Biotin<br>PE	7D4	BD	4.17µg/ml<br>0.83µg/ml
CD27	PECy7	LG.3A10	Biolegend	0.67µg/ml
CD31	FITC	MEC 13.3	BD	4.17µg/ml
CD38	APC	90	eBioscience	0.83µg/ml
CD44	Biotin<br>FITC<br>V450	IM7	BD<br>BD<br>BD	1.67µg/ml<br>0.67µg/ml<br>1.67µg/ml
CD45	APC<br>FITC<br>PE	30-F11	BD<br>BD<br>BD	0.67µg/ml<br>1.67µg/ml<br>0.83µg/ml
CD45.1	APC<br>Biotin<br>PerCPCy5.5	A20	eBioscience<br>Biolegend<br>eBioscience	0.67µg/ml<br>1.67µg/ml<br>1.67µg/ml
CD45.2	Biotin<br>FITC<br>PE<br>PECy7<br>PerCPCy5.5	104	BD<br>BD<br>BD<br>Biolegend<br>eBioscience	1.67µg/ml<br>1.67µg/ml<br>0.67µg/ml<br>0.83µg/ml<br>1.67µg/ml
CD69	PECy7	HI.2F3	BD	0.83µg/ml
CD90.2	APC	53-2.1	BD	0.56µg/ml
CD121α (IL-1R1)	PE	JAMA-147	Biolegend	3.33µg/ml
CD140α	BV421	APA5	BD	1.11µg/ml
Ep-CAM	Purified	G8.8	BD	2.08µg/ml
gp38	Biotin	eBio8.1.1	eBioscience	2.78µg/ml
I-A/I-E	Biotin	2G9	BD	2.08µg/ml
IFN-γ	Purified (cell culture)<br>FITC	XMG1.2	BioXCell<br>BD	10µg/ml<br>2.78µg/ml
IL-17A	BV421<br>BV510<br>PE	TC11-<br>18H10.1	Biolegend<br>Biolegend<br>BD	0.28µg/ml<br>1.33µg/ml<br>1.11µg/ml
IL-23R	APC	753317	R&D	20µL/well
Ly6G	FITC	1A8	BD	1.67µg/ml
RORγt	PerCPeFluor710	B2D	eBioscience	1.67µg/ml
Sca-1	FITC	D7	eBioscience	2.08µg/ml
TCR-γδ	Biotin<br>BV421<br>PECy7<br>Purified (cell culture)	GL3	eBioscience<br>Biolegend<br>Biolegend<br>Biolegend	1.67µg/ml<br>0.83µg/ml<br>0.83µg/ml<br>1µg/ml
Vγ4	PECy7	UC3-10A6	eBioscience	0.67µg/ml

Supplementary Table 1 (continued)				
**Other flow cytometry reagents**				
Reagent	Conjugate	Clone	Company	Final dilution
α-rat IgG	Alexa 647	Polyclonal	Life Technologies	10μg/ml
Proliferation dye	eFluor670	-	eBioscience	5μM
Live/Dead fixable dye	Near infrared	-	Life Technologies	1/1000
Streptavidin	Alexa 647<br>BV510<br>PerCPCy5.5	-	Jackson ImmunoResearch<br>BD	2.5μg/ml<br>0.33μg/ml<br>0.67μg/ml

Supplementary Table 2: Primers used in qPCR

Gene	Forward primer (5’→3’)	Reverse primer (5’→3’)
Rplp0	TGC AGA TCG GGT ACC CAA CT	ACG CGC TTG TAC CCA TTG A
Ccr2	GTT CAT CCA CGG CAT ACT ATC AAC	GCC CCT TCA TCA AGC TCT TG
Ccr6	CCT GGG CAA CAT TAT GGT GGT	CAG AAC GGT AGG GTG AGG ACA
Rorc	CAG CCA ACA TGT GGA AAA GCT	GGG AAG GCG GCT TGG A
Irf4	CGG GCA AGC AGG ACT ACA AT	ACA ATG CCC AAG CCT TGA TG
Irf8	GCT GAT CTG GGA AAA TGA TGA GA	CAC CTC CTG ATT GTA ATC CTG CTT
Prdm1	TGG CAG AGA CTG GGA TCA TG	CTC GGC CTC TGT CCA CAA A
Batf	GTT CTG TTT CTC CAG GTC C	GAA GAA TCG CAT CGC TGC
Tbx21	GCC AGG GAA CCG CTT ATA TG	AAC TTC CTG GCG CAT CCA
Eomes	TGA GCT TCA ACA TAA ACG GAC TCA	CGG CCA GAA CCA CTT CCA
